# Discontinuous boundaries of slow slip events beneath the Bungo Channel, southwest Japan

**DOI:** 10.1038/s41598-017-06185-0

**Published:** 2017-07-21

**Authors:** Ryoko Nakata, Hideitsu Hino, Tatsu Kuwatani, Shoichi Yoshioka, Masato Okada, Takane Hori

**Affiliations:** 1 Research and Development Center for Earthquake and Tsunami, Japan Agency for Marine-Earth Science and Technology, 3173-25, Showa-machi, Kanazawa-ku, Yokohama, Kanagawa 236-0001 Japan; 2 Department of Computer Science, University of Tsukuba, Tsukuba, Japan; 3 Department of Solid Earth Geochemistry, Japan Agency for Marine-Earth Science and Technology, Yokosuka, Japan; 4 PRESTO, Japan Science and Technology Agency, Kawaguchi, Japan; 5 Research Center for Urban Safety and Security, Kobe University, Kobe, Japan; 6 Graduate School of Frontier Sciences, The University of Tokyo, Kashiwa, Japan

## Abstract

The down-dip limit of the seismogenic zone and up-dip and down-dip limits of the deep low-frequency tremors in southwest Japan are clearly imaged by the hypocentre distribution. Previous studies using smooth constraints in inversion analyses estimated that long-term slow slip events (L-SSEs) beneath the Bungo Channel are distributed smoothly from the down-dip part of the seismogenic zone to the up-dip part of the tremors. Here, we use fused regularisation, a type of sparse modelling suitable for detecting discontinuous changes in the model parameters to estimate the slip distribution of L-SSEs. The largest slip abruptly becomes zero at the down-dip limit of the seismogenic zone, is immediately reduced to half at the up-dip limit of the tremors, and becomes zero near its down-dip limit. Such correspondences imply that some thresholds exist in the generation processes for both tremors and SSEs. Hence, geodetic data inversion with sparse modelling can detect such high resolution in the slip distribution.

## Introduction

In many subduction zones along the Pacific Rim, deeper extents of the source areas for megathrust earthquakes have recorded slow earthquakes in the past two decades^1^. Similar fault slips seem to induce both slow earthquakes and megathrust earthquakes in subduction zones^2^. However, slow earthquakes have much longer characteristic time scales (i.e., event duration) compared to regular earthquakes. In particular, the characteristic time scales are shorter and the magnitudes are smaller for deeper slow earthquakes in subduction zones. The mechanism of slow earthquakes has yet to be elucidated as a function of depth. To better understand these processes, it is important to investigate various types of slow earthquakes within the same region at different depths using precise seismic and geodetic observation data from several stations.

Several works have summarised details of slow earthquakes in many subduction zones^1, 3, 4^. Among the various types of slow earthquakes, well-studied events include deep low-frequency tremors^5^ with dominant frequency of 2–8 Hz, especially in the Nankai and Cascadia subduction zones. Seismic data indicates that the up-dip and the down-dip limits of deep tremors are sharply distributed within a narrow belt-like zone^5^. Once tremors occur, they usually continue for several days and accompany deep very low-frequency earthquakes (VLFs) at a dominant period of 10–100 s^6^. Short-term slow slip events (S-SSEs) typically have a duration of 2–6 days in the Nankai subduction zone^3, 7^ and approximately 10 days in the Cascadia subduction zone^8^. The association of tremors and S-SSEs is referred to as episodic tremor and slip (ETS)^8^. Because deep tremors occur along the isotherm contour and not the isodepth contour^9^ and they exhibit various migration properties, tremor generation processes have been related to fluids from the dehydration of materials in the subducting overriding plate, or both^5, 10, 11^. The sharp boundary of the tremor area seems to be consistent with a generation mechanism affected by chemical processes like dehydration onset at a certain temperature threshold.

**Figure 1:**
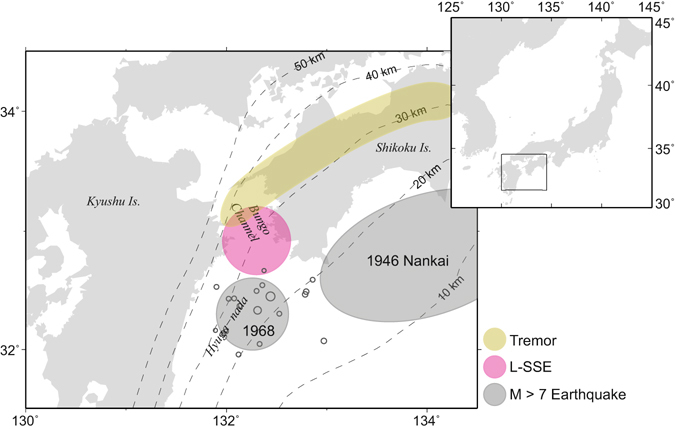
Distribution of various types of fault slips. Red circle approximates the source areas of L-SSEs. Grey circles are the source areas of the 1946 Nankai and the 1968 Hyuga-nada earthquakes. Yellow area approximates the source area of deep low-frequency tremors. Small circles represent hypocentres of earthquakes M > 3.0 and depth < 70 km from 1 April to 1 May 1968 as determined by the Japan Meteorological Agency (JMA). Contours indicate the depth (in km) of the upper surface of the descending Philippine Sea plate^30^. Characters in italics are geographical names. Rectangle in the inset represents the location of the study area. The map is created by Generic Mapping Tools software (GMT v4.5.12; http://gmt.soest.hawaii.edu/)^33^.

There is a spatial gap between megathrust earthquakes and deep ETSs. Long-term slow slip events (L-SSEs) with durations of 0.5–5 years^3^ and recurrence interval of >5 years occur within this gap of the Nankai subduction zone. In contrast, L-SSEs have not been identified in the Cascadia subduction zone. The L-SSEs beneath the Bungo Channel in southwest Japan (in the Nankai subduction zone) are a type of slow earthquake with a long time scale. L-SSEs in this region occurred repeatedly around 1997, 2003, and 2010. The slip areas of these three events are estimated to nearly coincide^12^, filling a spatial gap between deep ETS and seismogenic zones (Fig. 1).

The upper (up-dip) boundary of the L-SSEs area was located close to the sources of two M > 7 interplate earthquakes (the 1946 M 8.0 Nankai earthquake and the 1968 M 7.5 Hyuga-nada earthquake). This implies partitioning between seismogenic and aseismic slip areas as highlighted from slow slip events^13^. The lower (down-dip) boundary of the L-SSEs area partly overlaps with the source areas of deep tremors^12, 14^. Temporal correspondence has been reported between the occurrence of L-SSEs and the acceleration of deep tremors beneath the Bungo Channel^14^.

In contrast to the discontinuous boundary of the existing tremor area determined by seismic data, the total slip distributions of these L-SSEs estimated by geodetic data have been smoothed to some extent due to prior constraints on inversion analyses^12, 15^. Smoothness constraints to the spatial variation of fault slips have been widely used for geodetic data inversion^16, 17^. It is possible that the slip distribution of L-SSEs is similar to that of tremors but the smoothness constraints render the distribution smooth.

Recently, a sophisticated scientific information method using sparsity constraints^18, 19^, which is referred to as compressed sensing, has been applied to various natural science fields^20, 21^. In geophysics, sparsity constraints^18^ have been introduced as a priori information into geodetic inversions instead of the commonly used smoothness constraints^22, 23^. Sparsity constraints have shown compact and sharply varying slip distributions with discontinuous boundaries, which are close to the end-member image of a slip distribution and previously unimaginable. Consequently, we introduce sparsity constraints into the slip distribution analyses of L-SSEs beneath the Bungo Channel to explore the possible existence of sharply varying slip distributions with discontinuous boundaries.

## Data

We used digital data for the observed vertical and horizontal displacements of the 1997, 2003, and 2010 L-SSEs, which were in the Supporting Information of Yoshioka *et al*.^12^. Crustal displacements were observed at the positions of Global Navigation Satellite System (GNSS) stations operated by the Geospatial Information Authority of Japan. In all, we used 33, 65, and 106 continuous GNSS stations as the number of stations increased over time (Supplementary Figure 1). We used only the total displacement of each component during periods from 1996.7 to 1998.5, 2001.9 to 2004.5, and 2009.5 to 2011.2. In this study, we focused only on the spatial distribution to estimate the detailed distribution of the total slips during each L-SSE event.

## Results

**Figure 2:**
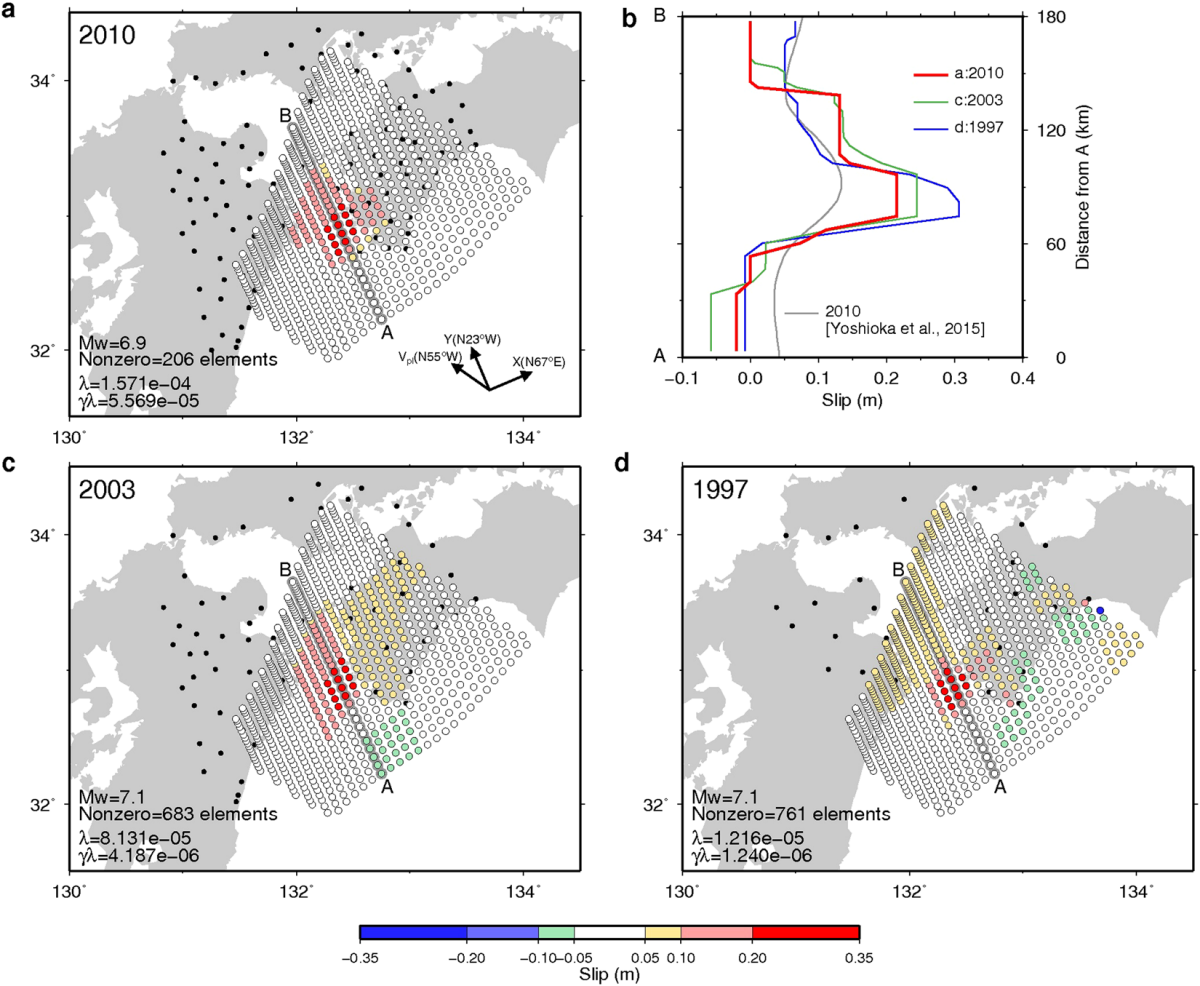
(**a**) Estimated slip distribution in the dip direction in the *X*–*Y* plane for the 2010 event. Black dots indicate GEONET stations. (**b**) Slip distribution along line AB. Blue, green, and red represent the 1997, 2003, and 2010 events, respectively. Grey line represents the 2010 event estimated by smoothed inversion^12^. (**c**) Estimated slip distribution in the *X*–*Y* plane for the 2003 event. (**d**) Estimated slip distribution for the 1997 event. The maps and graph are created by Generic Mapping Tools software (GMT v4.5.12; http://gmt.soest.hawaii.edu/)^33^.

The slip distribution on the plate interface was estimated using fused regularisation^24^ (see Methods). For simplicity, only dip-slips on the fault plane were considered. For the 2010 L-SSEs, slips > 0.2 m were concentrated to a relatively narrow area, and slips < 0.2 m were mainly distributed on the deeper side (Fig. 2a). There were no slips toward the shallower area (Fig. 2a). The cross section of the estimated spatial distribution of the slips along line AB showed a clear step at the down-dip side and a steep boundary at the up-dip side (Fig. 2b, red line). The 1997 and 2003 L-SSEs showed similar distributions to that of the 2010 L-SSE (Fig. 2c,d). However, due to fewer observation stations, the spatial resolutions were lower than that for 2010. Large (>0.2 m) slips were estimated to have occurred at almost the same locations identified by Yoshioka *et al*.^12^. However, the slip distributions at the up-dip side significantly differed from the previous study^12^.

The unique elements of our results are the up-dip boundary of the L-SSE area and the step-like distribution of the down-dip side. The moment magnitude (Mw), subfaults with nonzero slips, and selected values of the two hyperparameters in our estimation model are described in the lower left of Fig. 2a,c and d, and Table S1. The Mw values of the estimated slip for the 1997, 2003, and 2010 events were comparable to those determined by Yoshioka *et al*.^12^.

Numerical inversion tests were conducted to assess how efficiently the proposed method reproduced the original slip distribution from noise-overlapped synthetic displacement data (Supplementary Figures 2, 3, 5, 6, and 7). For a test with a step-like distribution, the estimated slip modestly reproduced the original distribution, but the step-width at the deeper side was narrower than that of the true slip (Supplementary Figure 2b, red line). In another test with a bell-like distribution, the estimated slip did not show step-like distributions (Supplementary Figure 3b, red line). For the test with narrower and larger slip areas than that of the step-like distribution, the spatial resolutions could be confirmed at least around the major slip area (Supplementary Figures 5, 6, and 7).

The proposed method tended to estimate smoother slip patterns and did not show artificial discontinuity during the evaluation with synthetic datasets. Hence, the estimated step-like distributions for the real dataset were quite reliable. Furthermore, we conducted another test using smooth fused regularisation (see Methods) with positivity constraints for the same step-like slip as mentioned above. Instead of the step-like distribution, the estimated slips showed a triangular distribution (Supplementary Figure 8).

We also conducted a dependency check of the observation noise for the step-like distribution (see Methods). For 1,000 patterns of estimated slips, the noise dependence was sufficiently low to recognise the existence of two sharp boundaries, although the maximum slips differed (Supplementary Figures 4a, 4b, and 4c). Figure 2b shows the transition mechanism of characteristic event type from megathrust earthquakes to slow earthquakes based on the slip boundary.

## Discussion

**Figure 3:**
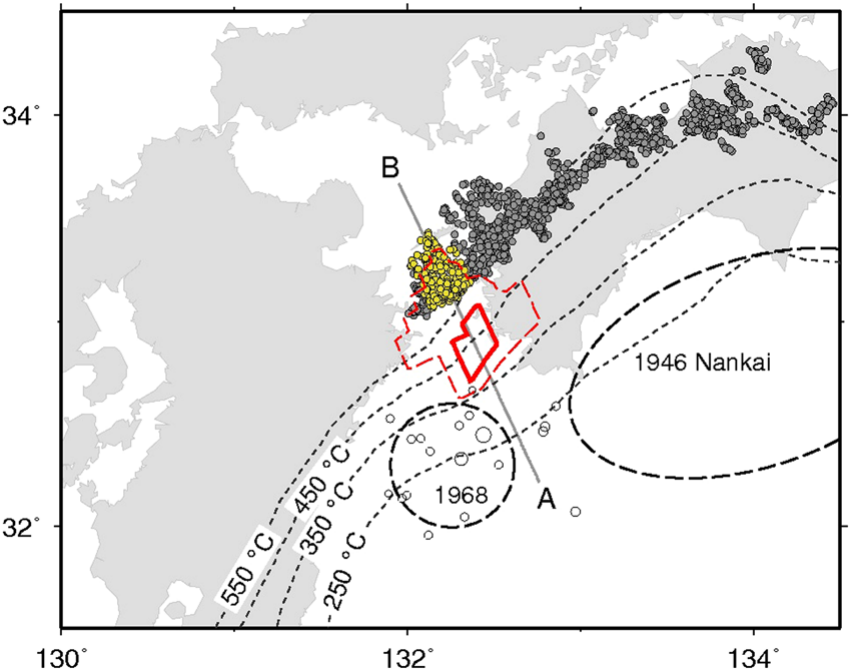
Red polygon with the solid and dashed lines shows the 2010 L-SSE with more than 0.2 m and 0.1 m slip areas estimated in this study. Yellow and grey circles indicate deep tremors detected during the 2010 L-SSE data period (2009.5 to 2011.2) using the modified envelope correlation method considering tremor amplitude^34^ and the hybrid-clustering method^35^ of the National Research Institute for Earth Science and Disaster Prevention (NIED) of Japan. Open ellipses with dashed lines indicate approximate source areas of the 1946 Nankai and 1968 Hyuga-nada earthquakes. Open circles represent hypocentres of earthquakes M > 3.0 and depth < 70 km from 1 April to 1 May 1968 as determined by JMA (Same as Fig. 1). Dashed contours show the temperature (250–550 °C) on the Philippine Sea plate^9^. The map is created by Generic Mapping Tools software (GMT v4.5.12; http://gmt.soest.hawaii.edu/)^33^.

**Figure 4:**
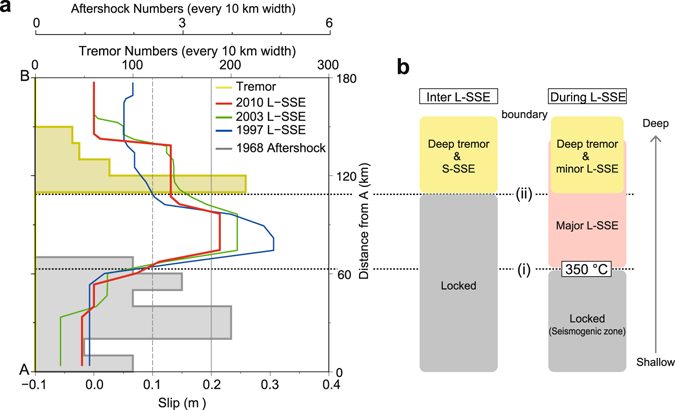
(**a**) Blue, green, and red lines represent slip distributions of the 1997, 2003, and 2010 L-SSEs along line AB (as in Fig. 2b). Yellow line shows the total tremor numbers in 10-km increments along line AB. Grey line shows the total number of aftershocks for the 1968 Hyuga-nada earthquake (in Figs 1 and 3) in 10-km increments along line AB. (**b**) Schematic of the spatial distribution of the locked zone, L-SSE, S-SSE, and deep tremors. The graph is created by Generic Mapping Tools software (GMT v4.5.12; http://gmt.soest.hawaii.edu/)^33^.

The L-SSE areas are clearly separated into two parts [the major (minor) slip area at the up-dip (down-dip) side] with two sharp boundaries (Figs 3 and 4). (i) The shallow boundary between the seismogenic zone and the major L-SSE area corresponds to the isotherm at 350 °C^9^. (ii) The deep boundary between the major L-SSE area and the minor L-SSE area corresponds to the up-dip limit of the tremor area.

In the area shallower than the up-dip limit of the major L-SSE area, a slip deficit accumulated^12^ and the 1968 Hyuga-nada earthquake (M7.5) occurred. The down-dip limit of the one-month aftershock of this earthquake did not penetrate into the L-SSE area (Fig. 3). The down-dip limit of the seismogenic zone and the up-dip limit of the SSE are clearly separated, and the boundary corresponds to the isotherm at 350 °C. Although it was previously noted that the down-dip limit of the seismogenic zone depends on temperature^25^, this is the first evidence that the up-dip limit of the SSEs also depends on temperature.

The correspondence between the up-dip limit of the tremors and that of the minor L-SSEs can be explained as follows (Fig. 4). In the inter L-SSE period, S-SSEs intermittently occur and tremors accelerate. Since part of the slip deficit is released during S-SSEs, the remaining slip deficit within the S-SSE area when L-SSEs occur is smaller than that of the major L-SSE area, where a tremor or S-SSE would not have occurred. On the other hand, during L-SSEs, S-SSEs also occur, accelerating tremors within the minor L-SSE area, as suggested by seismic tremor and geodetic SSE observations^14^.

The sharp variation in the slip amount of L-SSEs and the correspondence of the boundaries with up-dip limit of the tremor area suggest that frictional properties, which control slip behaviour, may change sharply. The onset of tremors corresponds to the dehydration process from metamorphic hydrous minerals, such as chlorite and epidote, in the subducting oceanic crust^26^. On the other hand, on fault surfaces like the plate boundary, frictional properties are not uniform. They are complex and include scale heterogeneity^27^. In particular, mixed brittle and ductile patches may be distributed in the area of slow earthquakes^28^. The ratio and connectivity of brittle and ductile patches may abruptly change at these boundaries.

Furthermore, it should be noted that sparse modelling can examine the extent that sharp edges, steps and small slip zones can fit the geodetic data, which only showed a smooth distribution in previous studies. Our results indicate that the resolution of the position of sharp edges estimated from geodetic data analysed with sparse modelling is comparable to the spatial resolution of the hypocentre distribution determined from seismic data. Such independent datasets of the spatial distribution are useful to hypothesise about the physical processes of both megathrust and slow earthquakes. Furthermore, a method based on the generalised fused regularisation should be available for any slip and slip-deficit distribution analyses on various types of faults, promoting the consideration of physical processes.

## Methods

### Geodetic inversion

The relationship between the displacement on a free surface and the slip on a plate interface is expressed as:

1 $${d}_{k}=\sum _{l=1}^{N}{G}_{kl}{s}_{l}+{\varepsilon }_{k}\,(k=1,2,\cdots ,K),$$

where *K* is the number of observed GNSS displacements. *d*_*k*_ is the observed displacements at the *k*-th station on the Earth’s surface. *s*_*l*_ is the dip-slip of the *l*-th subfault on the plate boundary. *N* is the number of subfaults. *G*_*kl*_ is Green’s function, which represents the displacement at station *k* due to a unit slip on subfault *l*. *ε*_*k*_ is the error (including observation noise) at the *k*-th station.

We divided the plate interface into small rectangular subfaults. Each subfault was approximated by three triangles to calculate the angular dislocation^29^. Green’s functions, which are the combined effect of three angular dislocations within a subfault in an elastic, homogeneous, and isotropic half space, were represented on a subfault. We used a realistic three-dimensional plate geometry^30^. The size of the rectangular subfaults was 9 km in the *X* direction (N 67° E). *N* is equal to 781 and the dip-slip only occurs for 781 subfaults. (For simplicity, we did not consider the strike-slip on the fault plane).

The number of unknown model parameters was greater than that of the observed parameters. Consequently, the above inversion problem is mathematically categorised as an ill-posed problem, which does not have a stable solution. To overcome this, most previous geodetic inversions have imposed smoothness constraints by adding the L2-reguralisation term, ${\sum _{i \sim j}|{s}_{i}-{s}_{j}|}^{2}$, to the term for reproducibility of the observation, ${\Vert {\boldsymbol{d}}-G{\boldsymbol{s}}\Vert }_{2}^{2}$, in the evaluation function. The L2-regularisation term acts as a smoother by decreasing the total gap of the slip between neighbouring subfaults. Occasionally, this term excessively smooths the distribution of slips, especially when amount and quality of observations are poor.

In this study, we used sparse modelling, which is a form of statistical analysis to solve a problem that introduces the sparsity of the solution as a priori information^31^. In our problem, there were no-slip areas, where the slip behaviours were a steady state within the assumed system of subfaults. Using generalised fused regularisation^24^, which is a kind of sparse modelling, we estimated the slip distribution as:

2 $${\hat{s}}^{\ast }=\mathop{{\rm{a}}{\rm{r}}{\rm{g}}{\rm{m}}{\rm{i}}{\rm{n}}}\limits_{{\boldsymbol{s}}}\{{\parallel {\boldsymbol{d}}-G{\boldsymbol{s}}\parallel }_{2}^{2}+\lambda \sum _{i\sim j}|{s}_{i}-{s}_{j}|+\gamma \cdot \lambda {\parallel {\boldsymbol{s}}\parallel }_{1}\},$$

where *λ* (≥ 0) is a regularisation parameter for fused smoothness and *γ* (≥ 0) is a hyperparameter to control the ratio between the fusion and the sparsity penalty terms. A large value of *γ* · *λ* decreases the non-zero components, while *γ* · *λ* = 0 introduces no sparsity. Σ_*i*~*j*_ is the summation of all pairs of neighbouring cells. The first term on the right-hand side of equation (2) represents the reproducibility between the model parameters and the observations. The second term represents the fused regularisation term, which indicates the smoothness and flatness of model parameters (***s***). The third term represents the sparsity constraint.

Optimal solutions were obtained using a set of hyperparameters with minimum residuals [mean squared residual (MSR), defined by $\sum _{k=1}^{K}{|{d}_{k}-\sum _{l=1}^{N}{G}_{kl}{s}_{l}|}^{2}/K$] via leave-one-out (LOO) cross validation^32^. In the LOO method, which is a model parameter estimation scheme, we used *N*−1 observation data to estimate the model parameters and one observation to calculate the MSR. We selected the values of the two hyperparameters by searching 200 different values of *γ*, ranging from 10 to 0. For the value of *γ*, we first took a large value of *λ*, which rendered all model parameters to 0. Then we took a smaller value of *λ*, which increased one nonzero model parameter. We iterated these calculations until *λ* = 0 or 4,000 times with decreasing *λ*.

### Numerical tests using synthetic data

Numerical inversion tests were conducted to assess how efficiently the proposed method reproduced the original slip distribution from the noise-overlapped synthetic displacement data. To assess the accuracy of our proposed method, we used synthetic displacements calculated from two end-member patterns of synthetic slips: a step-like distribution, including discontinuity, and a bell-like smooth distribution (Supplementary Figures 2a and 3a). For the step-like distribution, the synthetic slip consisted of 88 subfaults with nonzero slips (12 subfaults with 0.25 m and 76 subfaults with 0.10 m) and Mw = 6.874. These slip areas covered subfaults with more than 0.1-m slips estimated for at least one L-SSE. For the bell-like distribution, the synthetic slip consisted of 221 subfaults with nonzero slips (17 subfaults with 0.20–0.25 m, 38 subfaults with 0.10–0.20 m, and 24 subfaults with 0.05–0.10 m) and Mw = 6.929.

Similar to Nakata *et al*.^23^, we calculated the synthetic displacements on the free surface using the results from these synthetic slips. These were calculated using the same positions as Yoshioka *et al*.^12^ (Supplementary Figure 1). To represent the observation noise in the synthetic displacement data, we added independent random numbers that followed a normal distribution (mean = 0; standard deviations of east–west, north–south, and up–down components were 0.13 cm, 0.13 cm, and 0.56 cm for the 1997 L-SSE, 0.14 cm, 0.14 cm, and 0.57 cm for the 2003 L-SSE, and 0.14 cm, 0.14 cm, and 0.50 cm for the 2010 L-SSE, respectively). In these tests, we searched 40 different values of *γ*, ranging from 4 to 0, and referred to the results of the GEONET data. We iterated these calculations until *λ* = 0 or 1,000 times with decreasing *λ*.

The noise dependency check was conducted using 1,000 sets of noise-overlapping displacement data for the step-like synthetic slip (Supplementary Figures 4a, 4b, and 4c). We used the same values of the two hyperparameters (*λ* and *γ*) as those obtained in the first calculation (shown in Supplementary Figure 2) for all 1,000 estimations.

To investigate resolution of the spatial distribution, we conducted three numerical experiments based on the step-like slip (Supplementary Figures 5, 6, and 7). One removed subfaults with a 0.1-m slip on the east-west side of the large slip (Supplementary Figure 5a). The synthetic slip consisted of 42 subfaults with nonzero slips (12 subfaults with 0.25 m and 30 subfaults with 0.10 m) and Mw = 6.709. The second removed subfaults on the deep side (Supplementary Figure 6a). The synthetic slip consisted of 43 subfaults with nonzero slips (12 subfaults with 0.25 m and 31 subfaults with 0.10 m) and Mw = 6.714. The last one added subfaults on the shallow side (Supplementary Figure 7a). The synthetic slip consisted of 121 subfaults with nonzero slips (12 subfaults with 0.25 m and 109 subfaults with 0.10 m) and Mw = 6.952.

Furthermore, we applied smooth fused regularisation, which replaced the second term in equation (2) by L2 norm (equation (3)) for a step-like slip (Supplementary Figure 8). In this method, the hyperparameters were conventionally defined in a slightly different way in terms of *γ* and *λ*. Then in the case of the stations for the 2010 L-SSE, we tested seven values of *λ* ( = 0, 1.06, 2.12, 10.6, 21.2, 106, and 212) and searched *γ* for up to 1,000 times. The cross validation indicated that *λ* = 10.6 showed the minimum MSR for the 2010 stations.

3 $${\hat{s}}^{\ast }=\mathop{{\rm{a}}{\rm{r}}{\rm{g}}{\rm{m}}{\rm{i}}{\rm{n}}}\limits_{{\boldsymbol{s}}}\{{\parallel {\boldsymbol{d}}-G{\boldsymbol{s}}\parallel }_{2}^{2}+\lambda \sum _{i\sim j}{({s}_{i}-{s}_{j})}^{2}+\gamma {\parallel {\boldsymbol{s}}\parallel }_{1}\}.$$

## Electronic supplementary material

Supplementary Information
