# Supplementary material for: Discontinuous boundaries of slow slip events beneath the Bungo Channel, southwest Japan
Source: Sci Rep. 2017 Jul 21;7:6129. doi: 10.1038/s41598-017-06185-0 (PMC5522493; doi:10.1038/s41598-017-06185-0)
Supplement: Supplementary file 1 — Supplementary Information [file 41598_2017_6185_MOESM1_ESM.pdf]

Supplementary information

Discontinuous boundaries of slow slip events beneath the Bungo Channel, southwest Japan

Ryoko Nakata[1]*, Hideitsu Hino[2], Tatsu Kuwatani[3,4], Shoichi Yoshioka[5], Masato Okada[6], Takane Hori[1]

[1]Research and Development Center for Earthquake and Tsunami, Japan Agency for Marine-Earth Science and Technology, 3173-25, Showa-machi, Kanazawa-ku, Yokohama, Kanagawa 236-0001, Japan, *nakatar@jamstec.go.jp; [2]Department of Computer Science, University of Tsukuba, Japan; [3]Department of Solid Earth Geochemistry, Japan Agency for Marine-Earth Science and Technology, Japan; [4]PRESTO, Japan Science and Technology Agency, Japan; [5]Research Center for Urban Safety and Security, Kobe University, Kobe, Japan; [6]Graduate School of Frontier Sciences, The University of Tokyo, Japan

Table S1 Slip-related values for the 1997, 2003, and 2010 L-SSEs estimated from inversion analysis of displacement data from Yoshioka et al. [2015].

	2010 L-SSE	2003 L-SSE	1997 L-SSE
Mw	6.904	7.127	7.073
Maximum amount of slip	0.214	0.245	0.306
Subfaults with nonzero slip	206	683	761
Hyperparameter λ	1.571e-04	8.131e-05	1.216e-05
Hyperparameter γλ	5.569e-05	4.187e-06	1.240e-06

Supplementary Figures and Figure legends

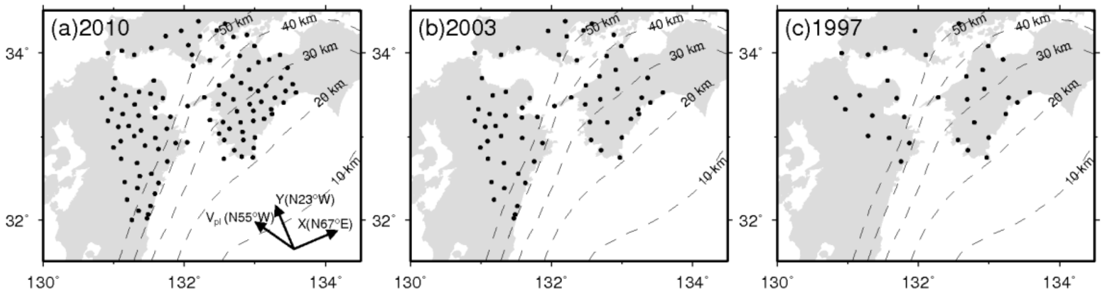

Supplementary Figure 1 Black dots represent GEONET stations used in this study. The maps were created using Generic Mapping Tools software (GMT v4.5.12; http://gmt.soest.hawaii.edu/)[30].

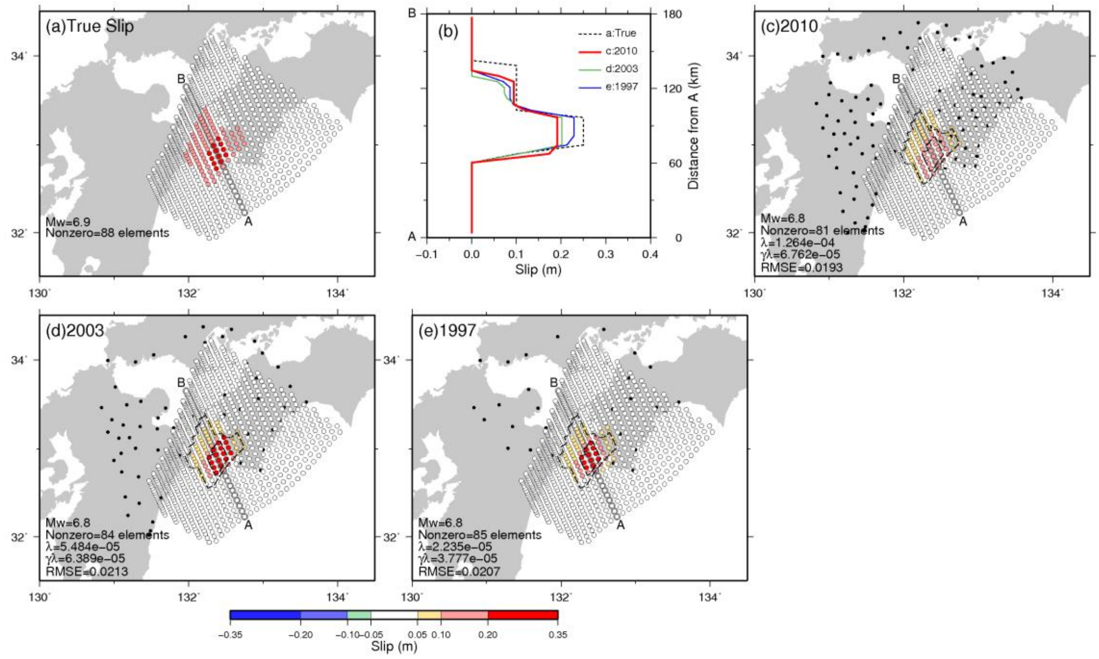

Supplementary Figure 2 Slip distributions on the plate interface estimated from synthetic displacement data with random noise from a step-like slip. **(a)** True slip distribution in the *X*–*Y* plane. **(b)** Profile along line AB. Blue, green, and red lines represent the 1997, 2003, and 2010 events, respectively. **(c)**–**(e)** Estimated slip distribution using stations for the 1997, 2003, and 2010 L-SSEs. Black dots represent GEONET stations. The maps and graph were created using Generic Mapping Tools software (GMT v4.5.12; http://gmt.soest.hawaii.edu/)[30].

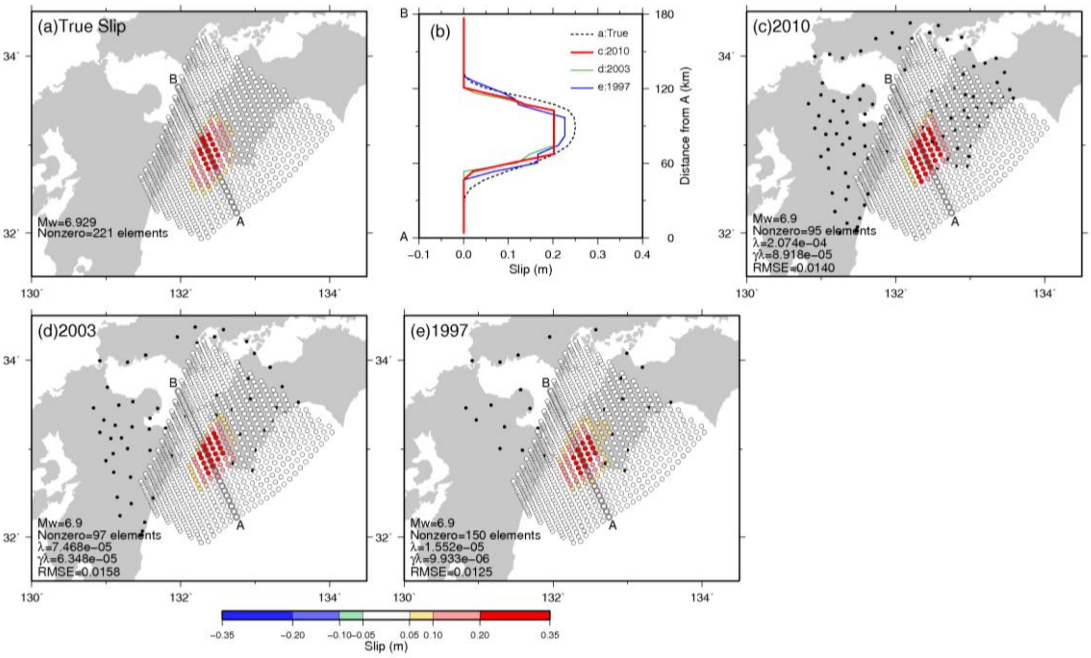

Supplementary Figure 3 Slip distributions on the plate interface estimated from synthetic displacement data with random noise from a bell-like slip. **(a)** True slip distribution in the *X*–*Y* plane. **(b)** Profile along line AB. Blue, green, and red lines represent the 1997, 2003, and 2010 events, respectively. **(c)**–**(e)** Estimated slip distribution using stations for the 1997, 2003, and 2010 L-SSEs. Black dots represent GEONET stations. The maps and graph were created using Generic Mapping Tools software (GMT v4.5.12; http://gmt.soest.hawaii.edu/)[30].

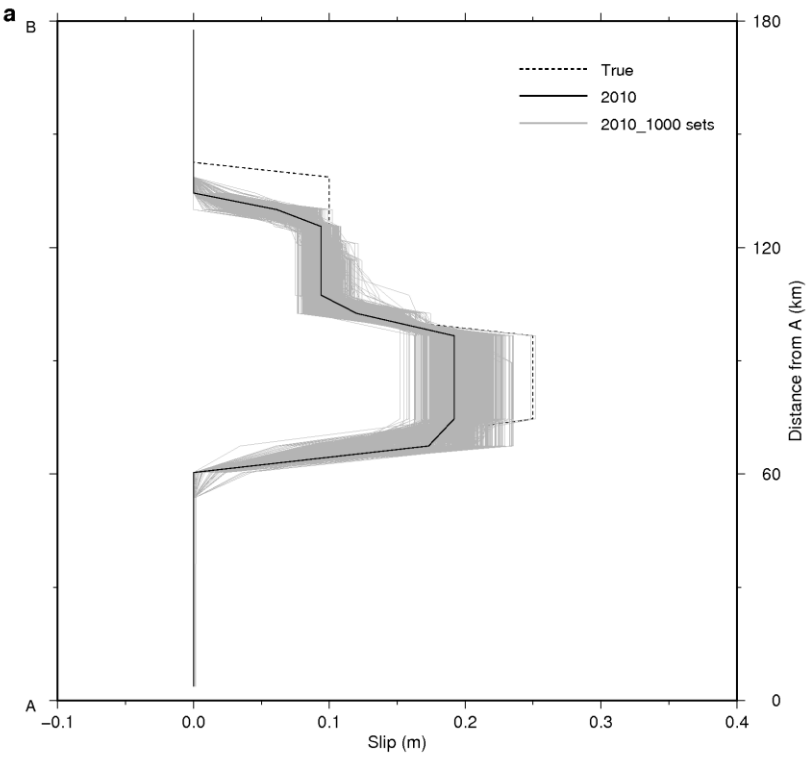

a
B
A
True
2010
2010_1000 sets
−0.1
0.0
0.1
0.2
0.3
0.4
Slip (m)
0
60
120
180
Distance from A (km)

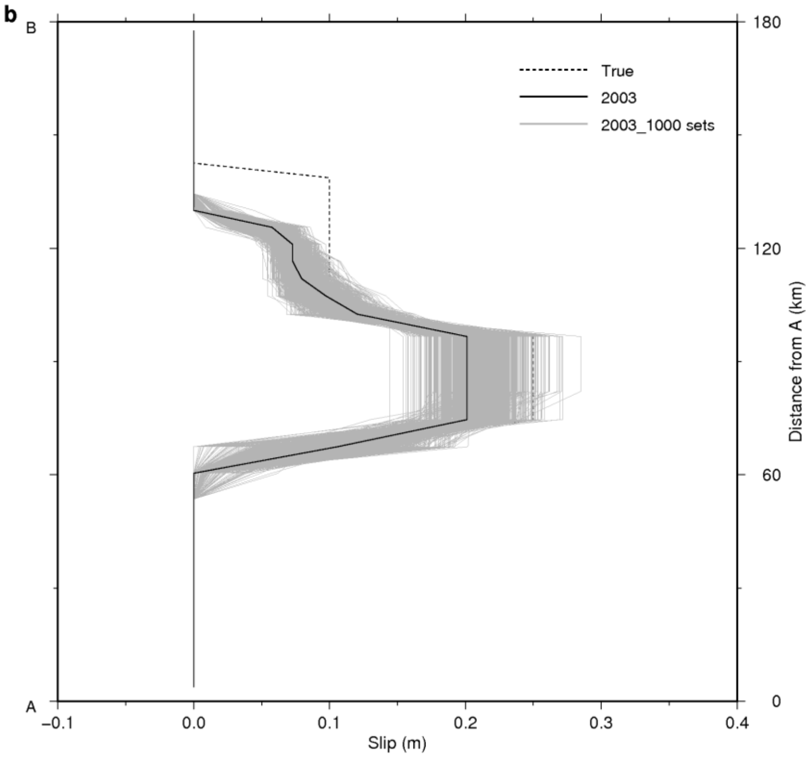

b
B
A
True
2003
2003_1000 sets
−0.1
0.0
0.1
0.2
0.3
0.4
Slip (m)
0
60
120
180
Distance from A (km)

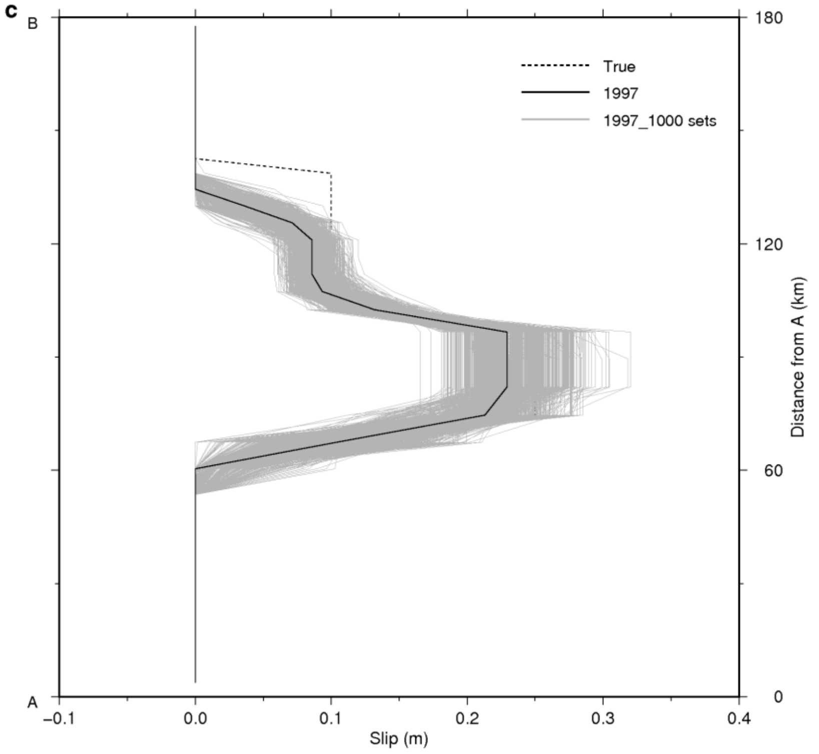

Supplementary Figure 4 (a) Slip profile along line AB. Grey lines indicate estimated slips from 1,000 sets of noise-overlapped synthetic displacements from 106 stations for the 2010 L-SSEs. **(b)** Slip profile along line AB (as in Supplementary Figure 4a). Grey lines indicate estimated slips using the stations for the 2003 event. **(c)** Slip profile along line AB (as in Supplementary Figure 4a). Grey lines indicate the estimated slips using stations for the 1997 event. The graphs were created using Generic Mapping Tools software (GMT v4.5.12; http://gmt.soest.hawaii.edu/)[30].

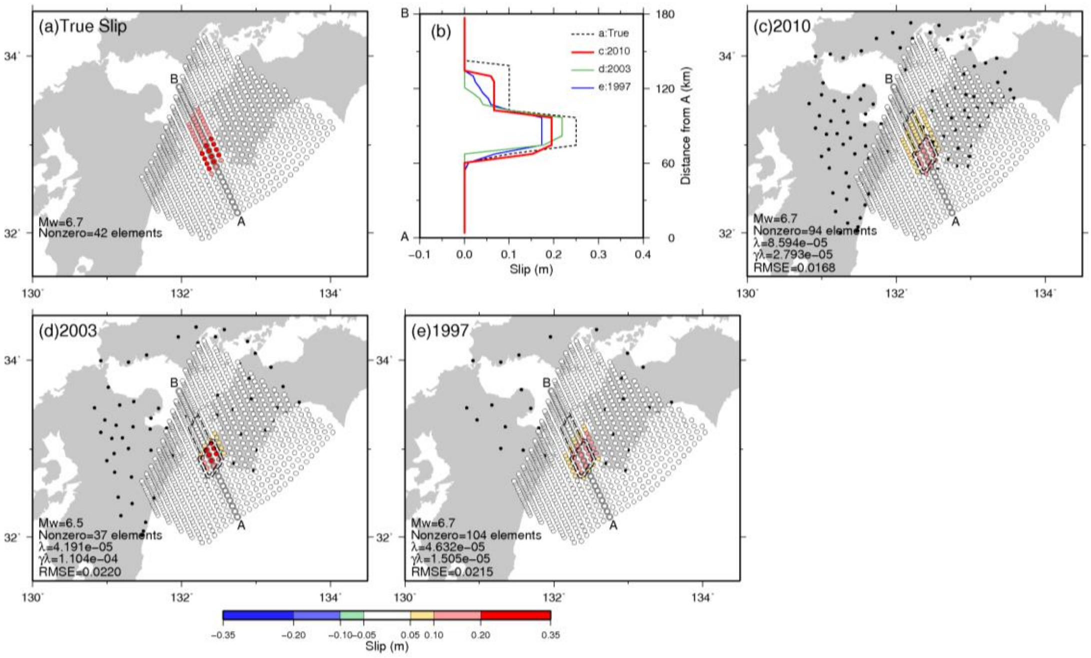

Supplementary Figure 5 Slip distributions on the plate interface estimated from synthetic displacement data with random noise from a step-like slip without slips on the east-west sides. **(a)**–**(e)** Same as those of Supplementary Figure 2. The maps and graph were created using Generic Mapping Tools software (GMT v4.5.12; http://gmt.soest.hawaii.edu/)[30].

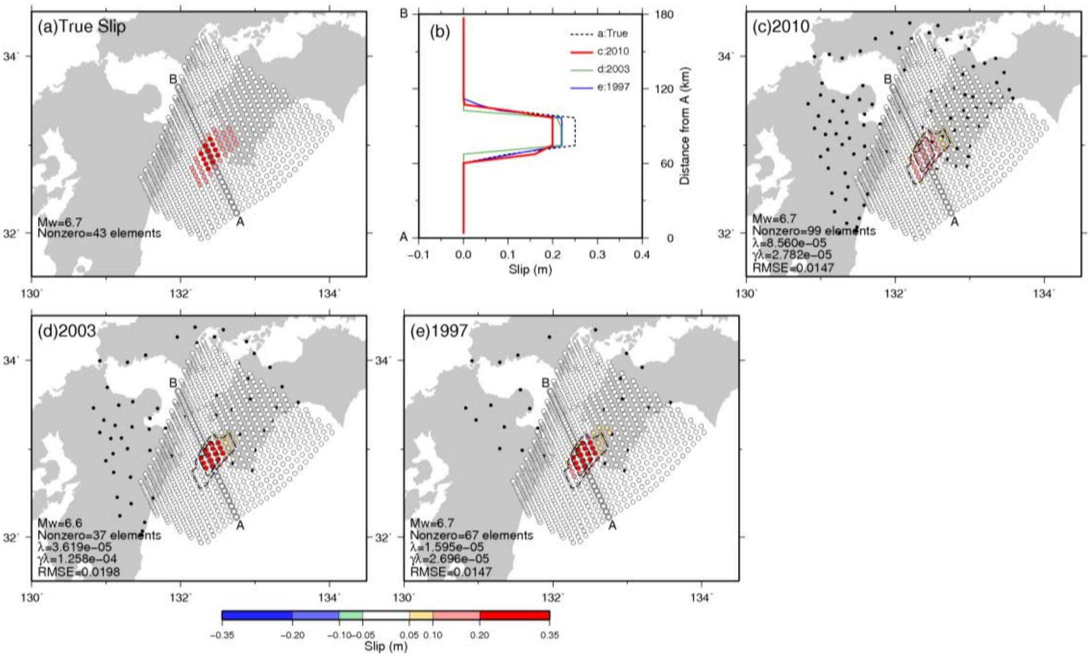

Supplementary Figure 6 Slip distributions on the plate interface estimated from synthetic displacement data with random noise from a step-like slip without slips on the deep sides. **(a)**–**(e)** Same as those of Supplementary Figure 2. The maps and graph were created using Generic Mapping Tools software (GMT v4.5.12; http://gmt.soest.hawaii.edu/)[30].

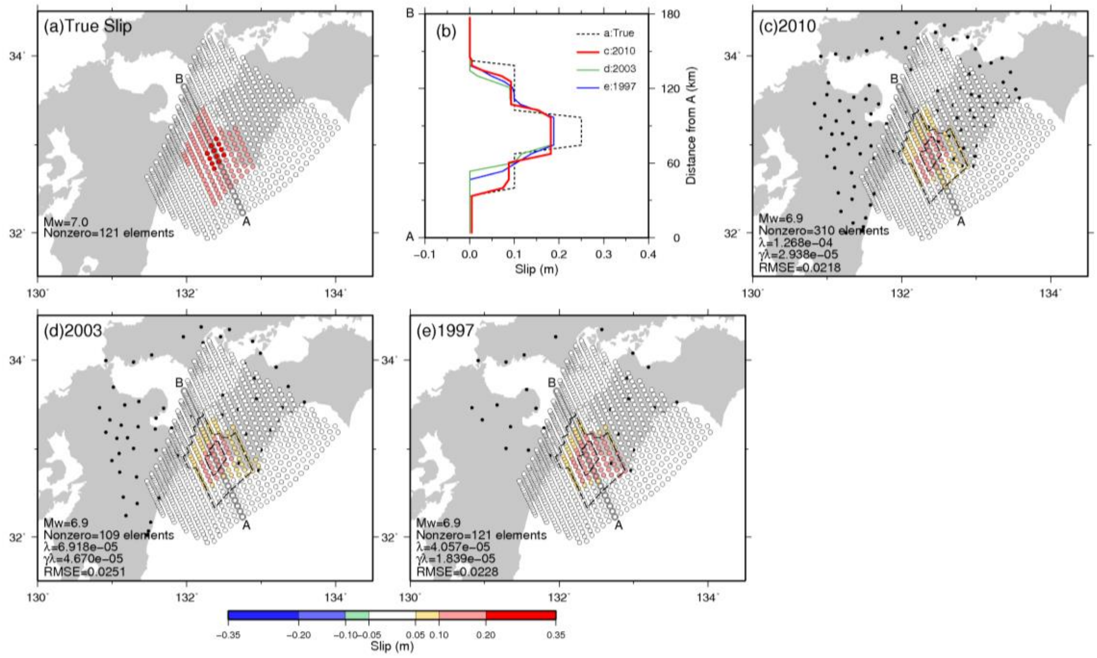

Supplementary Figure 7 Slip distributions on the plate interface estimated from synthetic displacement data with random noise from a step-like slip at both shallow and deep sides. **(a)**–**(e)** Same as those of Supplementary Figure 2. The maps and graph were created using Generic Mapping Tools software (GMT v4.5.12; http://gmt.soest.hawaii.edu/)[30].

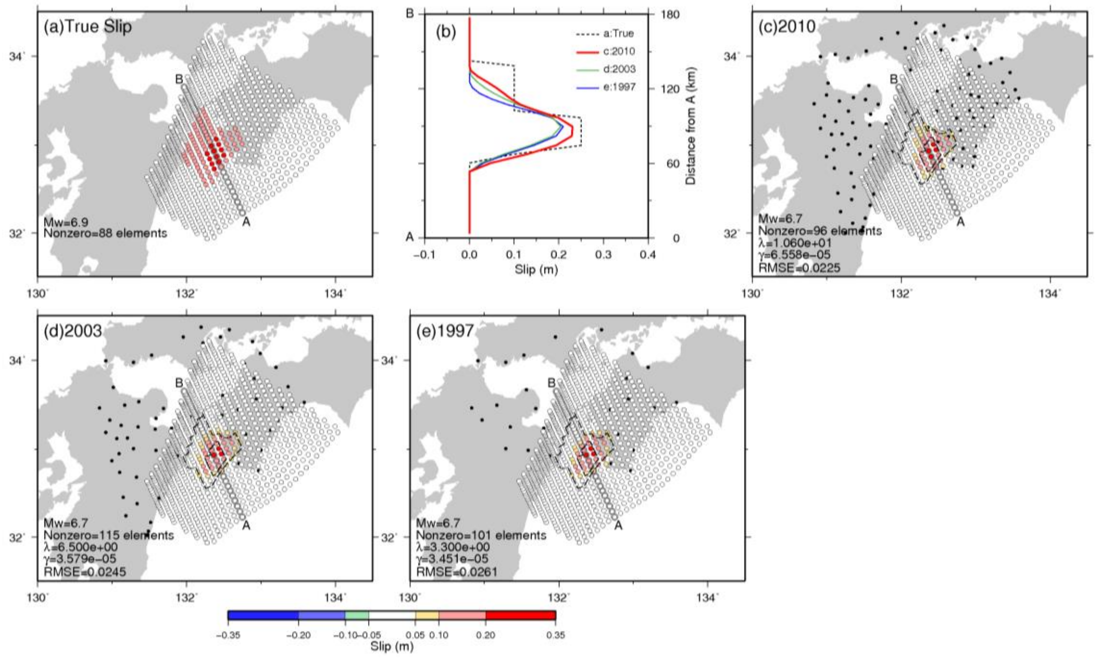

Supplementary Figure 8 Slip distributions on the plate interface estimated from synthetic displacement data with random noise from a step-like slip using smooth fused regularisation as the evaluation function. **(a)**–**(e)** Same as those of Supplementary Figure 2. The maps and graph were created using Generic Mapping Tools software (GMT v4.5.12; http://gmt.soest.hawaii.edu/)[30].
